# 

**Published:** 1888-12-29

**Authors:** 


**PERMANENTLY ENLARGED TO 32 PAGES.**

PRICE ONE PENNY.

No. 118, Vol. V.—December 29th, 1888.

[Registered at the General Post Office as a Newspaper.]

Per Annum, 6s. 6d., post free.

Monthly Parts. 6d.; post free, 7½d.

Ditto per Ann. 7s. 6d., post free.

### THE HOSPITAL

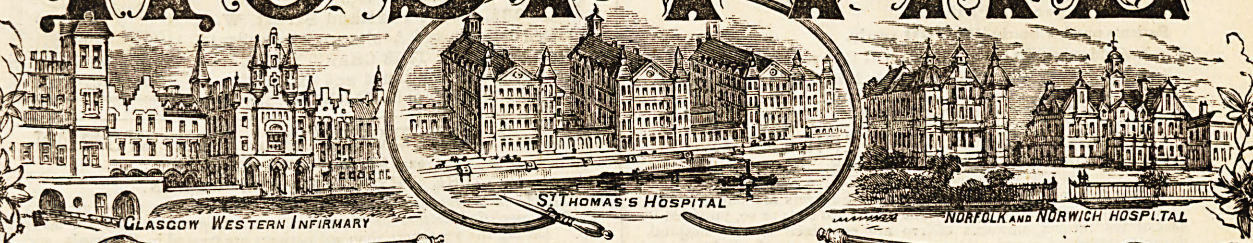

A WEEKLY INSTITUTIONAL JOURNAL OF

### Science, Medicine, Nursing, and Philanthropy.

### SPECIAL NEW YEAR'S NUMBER.

